# A Review of the Biotechnological Potential of Cave Fungi: A Toolbox for the Future

**DOI:** 10.3390/jof11020145

**Published:** 2025-02-14

**Authors:** Renan N. Barbosa, Maria Tamara C. Felipe, Leticia F. Silva, Edna A. Silva, Sabrina A. Silva, Polyanna N. Herculano, José F. S. A. Prazeres, Joenny M. S. Lima, Jadson D. P. Bezerra, Keila A. Moreira, Oliane M. C. Magalhães, Cristina M. Souza-Motta

**Affiliations:** 1 Departamento de Micologia, Universidade Federal de Pernambuco, Recife 50740-600, Pernambuco, Brazilfredson.alves@ufpe.br (J.F.S.A.P.); joenny.lima@ufpe.br (J.M.S.L.);; 2 Programa de Pós-Graduação em Biologia de Fungos, Departamento de Micologia, Universidade Federal de Pernambuco, Recife 50670-901, Pernambuco, Brazil; 3 Instituto de Patologia Tropical e Saúde Pública, Universidade Federal de Goiás, Goiânia 74605-050, Goiás, Brazil; 4 Departamento de Medicina Veterinária, Universidade Federal do Agreste de Pernambuco, Garanhuns 55292-270, Pernambuco, Brazil

**Keywords:** biotechnological applications, funga, fungal biotechnology, fungal metabolites, speleomycology

## Abstract

The study of the intersection between biodiversity and biotechnology has revealed a rich source of innovations. Fungi, with their vast range of morphologies and lifestyles, thrive in various habitats, including caves. With impressive metabolic characteristics, they play a key role in producing essential biotechnological compounds for various economic sectors. This paper aims to consolidate evidence on the biotechnological potential of fungi isolated from caves, highlighting the urgency of conserving and exploring these ecosystems. For this purpose, we conducted a comprehensive literature search using scientific databases (SciELO, Medline Complete, Medline/PubMed, Web of Science, Scopus (Elsevier), and Google Scholar). We adopted an interdisciplinary approach by collecting information from 22 papers published between 2013 and 2024. Based on these data, our survey revealed broad potential, including antimicrobial compounds, antioxidants, antitumor agents, enzymes, and organic acids. We emphasize that accurately identifying and depositing fungal isolates in reference collections are crucial for reliable research and effective industrial applications, driving metabolic bioactivity and the production of substances with the potential to inhibit pathogens. Conserving and protecting the cave environment is imperative, considering its continuous potential for discovery and contribution to scientific advancement.

## 1. Introduction

The intersection of biodiversity and biotechnology has emerged as a rich source of innovations [1,2,3]. Biodiversity, the cornerstone of life on Earth, is crucial for sustaining ecosystems and supporting our existence as a species. Additionally, it provides indispensable services to humanity, including vital contributions to medicine and healthcare [4]. For example, biodiversity serves as the primary source of pharmacologically active biomolecules, which are essential for discovering and developing new medications [5].

Fungi, the second largest group of organisms after insects [6], exhibit a wide range of morphologies, lifestyles, and developmental patterns and thrive in various habitats, including soil, water, air, plants, animals, and extreme environments [7,8]. Earth’s ecosystems are estimated to host between 1.5 and 12 million fungal species, with approximately 160,000 species formally described [9,10,11,12,13]. With impressive metabolic characteristics attributed to their intricate genomic networks, fungi are vital for producing biotechnological compounds essential in the pharmaceutical, food, feed, chemical, and biotechnology industries [14,15].

Fungi inhabit a wide variety of habitats and must compete against a diverse range of other fungi, bacteria, plants, and animals; thus, they have evolved numerous survival mechanisms [16] and exhibit unique adaptations in response to various extreme environments [17,18], resulting in a wealth of bioactive compounds and enzymes with potential applications in various biotechnological sectors [19,20,21]. Among the less-explored and extreme environments, caves and their inhabitants, especially microorganisms, are promising reservoirs of valuable biotechnological resources [22,23].

Extreme environments are habitats where temperatures, radiation, salinity, and pH reach values far outside the range considered normal for human life. These environments often lack macro life forms and are predominantly inhabited by highly adapted microorganisms, including fungi [17]. Notably, caves, which are mostly dark, nutrient-limited ecosystems with a constant temperature, high humidity, and low oxygen, provide an ideal example of such extreme environments [22].

Although the study of cave fungi dates back to 1794 when Humboldt first recorded unusual underground “plants” now known as fungi in Germany [24], cave research primarily focused on fauna and fungal pathogens like *Histoplasma capsulatum* and *Trichophyton mentagrophytes* until 1980 [25,26]. Since then, the focus has shifted toward the rich diversity of fungi in cave environments, and recent studies have shown an increasing interest in their biotechnological potential. A global review documented that 1626 species in 644 genera of fungi have been reported from caves and mines worldwide [27]. Caves are emerging as hotspots of microbial diversity, harboring both known and yet-to-be-discovered species, primarily from the phyla *Ascomycota*, *Basidiomycota*, and *Mucoromycotina* [24]. Recent studies conducted in Brazil have highlighted the diverse habitats of fungi, with significant findings on their presence in various ecological niches. These fungi inhabit various niches, including air, rocks, sediments, soil, water, guano, and organic matter [28,29,30,31,32,33,34].

Throughout history, the connection between humans and caves has been as old as human history itself, and they were historically used for various purposes, such as shelters and old sanctuaries [22,35], and have also been used for the treatment of patients with respiratory tract infections, a practice known as speleotherapy [36,37]. Caves harbor a largely unexplored microbial realm and present a potential reservoir of biological resources for antimicrobial and anticancer compounds. In particular, the oligotrophic conditions within caves promote the development of distinctive antimicrobial agents, which are essential for discovering new treatments in competitive microbial niches [22].

In response to the high demand for new medicines, cave ecosystems have long been investigated in the search for microorganisms, primarily Actinobacteria and other bacteria, with the potential to produce bioactive and antimicrobial compounds [38,39,40]. As the most predominant group of microorganisms in cave ecosystems, Actinobacteria species are a central focus in studies related to bioactive compounds and are recognized as a source of new antibiotics [41]. Actinomycetes derived from caves can produce highly complex compounds with biological activity. The specific characteristics of these compounds may vary depending on the culture media and methodology employed in the study [42]. For fungi, *Penicillium* isolated from cave sediments in Algeria demonstrated significant antimicrobial activity, inhibiting the growth of *Candida albicans* by more than 40% and reducing the growth of the bacteria *Micrococcus luteus* and *Staphylococcus aureus* by more than 30% [43].

Due to their oligotrophic conditions, caves harbor a unique diversity of organisms, including fungi adapted to extreme conditions, whose adaptations may lead to significant biotechnological discoveries. The isolation and preservation of these fungi in culture collections are crucial for providing samples with defined characteristics, which are essential for the development of new biotechnological processes and compounds. This review aims to consolidate studies published between 2013 and 2024, providing an overview of the biotechnological potential of cave fungi and offering insights into their industrial and pharmaceutical applications.

## 2. Materials and Methods

In this study, we conducted a comprehensive survey of relevant literature published between 2013 and 2024, focusing on the biotechnological potential of fungi isolated from caves around the world. Our search included databases such as SciELO Citation Index, Medline Complete, Medline/PubMed, Web of Science, Scopus (Elsevier), and Google Scholar, using the following search terms combined with the Boolean operator (AND): “cave fungi” and “biotechnology,” “cave fungi” and “enzymes,” “cave fungi” and “pigments,” “cave fungi” and “acid production,” “cave fungi” and “antitumoral compounds”, and “cave fungi” and “antimicrobial compounds.”

We limited our research to the Portuguese, English, and Spanish languages. The selection process occurred in two phases. Initially, we screened the titles and abstracts of all references to identify papers that potentially met our inclusion criteria. We do not consider dissertations, theses, or conference abstracts. In the subsequent phase, we thoroughly examined the full texts of the remaining articles, eliminating those that did not align with our goal. We extracted pertinent information from the retained articles, including author(s) names, publication year, cave name, country, fungal species, strain voucher, fungal identification method, and applications.

## 3. Results

Among the 22 publications that met our criteria, a total of 137 strains belonging to 22 genera were evaluated for their biotechnological potential. These genera include *Acremonium*, *Alternaria*, *Aspergillus*, *Beauveria*, *Bjerkandera*, *Botryotrichum*, *Botrytis*, *Cladosporium*, *Conidiobolus*, *Coprinellus*, *Epicoccum*, *Gliomastix*, *Lecanicillium*, *Mortierella*, *Parengyodontium*, *Penicillium*, *Pseudogymnoascus*, *Purpureocillium*, *Scopulariopsis*, *Stereum*, *Talaromyces*, and *Trichoderma*. A total of 48 species of filamentous fungi, along with some unidentified yeasts, were included in the studies.

Among these, *Aspergillus* and *Penicillium* species were the most frequently evaluated across various biotechnological applications. Specifically, *Aspergillus niger*, *A. flavus*, *A. fumigatus*, *A. sydowii*, and *A. tamarii* were the most commonly studied species. *Penicillium* also appeared prominently in this research, with multiple subspecies such as *Penicillium flavigenum*, *P. expansum*, and *P. samsonianum* being frequently mentioned in different cave studies.

Brazil stands out as the country with the highest number of investigations and caves examined. In these studies, *Aspergillus* and *Penicillium* species dominated, underscoring their relevance in biotechnological research. Notably, *Penicillium* was represented by a diverse array of subspecies across multiple Brazilian caves, indicating the country’s rich microbial diversity and its significant contribution to biotechnological exploration in this field (Table 1).

Although the number of studies on cave fungal diversity is increasing, much remains to be understood. Caves stand out as biodiversity hotspots, providing not only new fungal species but also those with unique metabolic activities not found in other environments [44,45,46], highlighting their importance for innovative advances in various areas of biotechnology. Several studies have investigated the fungal species in caves worldwide, highlighting the fact that they have a variety of applications. For example, Melo et al. [47] recorded diverse *Aspergillus* and *Penicillium* strains in the Brazilian caves using morphological identification, and these fungi were also related to tannase production. In the same way, Ogórek et al. [48] found *Penicillium glandicola* in Driny Cave in Slovakia and discussed its extracellular enzyme production potential. In Brazil, Paula et al. [49] dealt with the cellulase production by *Aspergillus* species from Gruta do Catão, while Souza et al. [50] investigated pigment production from the fungi *Aspergillus sydowii* (CML2967), *Aspergillus aureolatus* (CML2964; E.4.1), and *Lecanicillium aphanocladii* (CML2970) from Gruta dos Morcegos. The studies analyzed revealed biotechnological possibilities of cave-dwelling fungi ranging from enzyme production to antimicrobial and anticancer activities, which demonstrate the crucial role of cave ecosystems in biotechnological research.

**Table 1:** Cave fungi species, identification methods, and their biotechnological applications, published between 2013 and 2024.

Country	Name of the Cave	Fungal Species and Isolate/Strain Code	Identification Method	Applications	Reference
Brazil	Several caves: Garrincho, Moendas, Coroa de Frade, Cavalinho, Dimas, Tirrafa, Vila Corumbá, Olhos d’água, and Zé da Fazenda	*A. japonicus* (238, 246A, 260, 346A, 323, and 111), *A. foetidus* (35 and AT02), *A. niger* (37, 6, and 2A), *A. ochraceus* (17), *A. tamarii* (3, 53, and 127), *A. tubingensis* (588), *Aspergillus* sp. (47, GM4, and 9), *P. citrinum* (185B), *P. corylophillum* (493 and 92), *P. funiculosum* (302, 340, 718, 341, 289, and 247), *P. oxilacum* (21), and *Penicillium* sp. (273, 330, and 121)	Morphology	Tannase production	[47]
Slovakia	Driny Cave	*Penicillium glandicola* (UWR_002)	ITS (nBLAST) and morphology	Extracellular enzymes (amylases, proteases, cellulases, and pectinases)	[48]
Brazil	Gruta do Catão	*Aspergillus* sp. (SDC 1.1, SDC 1.2, SDC 1.4, SDC 1.6, SDC 2.4, SDC 2.6, SDC 2.8, SDC 2.10, SDC 2.11, and SDC 2.12), *Penicillium* sp. (SDC 1.3, SDC 1.5, SDC 2.7, SDC 2.9, and SDC 2.13), *Scopulariopsis* sp. (SDC 2.1), *Talaromyces* sp. (SDC 2.2), and *Purpureocillium* sp. (SDC 2.5)	Morphology	Cellulase production	[49]
Brazil	Gruta dos Morcegos	*Aspergillus sydowii* (CML2967)	Phylogenetic analysis based only ITS region	Pigment production	[50]
	Gruta Lapa Nova	*Aspergillus aureolatus* (CML2964 and E.4.1)			
	Gruta do Cavalinho	*Lecanicillium aphanocladii* (CML2970)			
Brazil	RM 3 cave	*Aspergillus candidus* (UFMGLMCC03, UFMGLMCC014, and UFMGLMCC028), *Aspergillus flavus* (UFMGLMCC05 and UFMGLMCC027), *Aspergillus fumigatus* (UFMGLMCC01 and UFMGLMCC13), and *Aspergillus niger* (UFMGLMCC016 and UFMGLMCC13)	Morphology (previous study by the same author)	Susceptibility to amphotericin B (AMB), itraconazole, voriconazole, and terbinafine	[51]
	Gruta do Bloco Abatido	*Aspergillus candidus* (UFMGLMCC011), *Aspergillus fumigatus* (UFMGLMCC07), and *Aspergillus terreus* (UFMGLMCC031)			
	Gruta do Cascalhinho	*Aspergillus candidus* (UFMGLMCC015), *Aspergillus flavus* (UFMGLMCC024), and *Aspergillus fumigatus* (UFMGLMCC010)			
	Gruta do Eremita	*Aspergillus flavus* (UFMGLMCC04), *Aspergillus niger* (UFMGLMCC018), and *Aspergillus niger* (UFMGLMCC023)			
	Gruta da Macumba	*Aspergillus flavus* (UFMGLMCC06) and *Aspergillus niger* (UFMGLMCC034)			
	Gruta dos Romeiros	*Aspergillus flavus* (UFMGLMCC09) and *Aspergillus tamarii* (UFMGLMCC020)			
	Gruta N. S. Conceição	*Aspergillus flavus* (UFMGLMCC019, UFMGLMCC021, UFMGLMCC030, and UFMGLMCC033), *Aspergillus fumigatus* (UFMGLMCC02 and UFMGLMCC022), *Aspergillus niger* (UFMGLMCC029), and *Aspergillus tamarii* (UFMGLMCC012)			
	RM 2 cave	*Aspergillus flavus* (UFMGLMCC025), *Aspergillus niger* (UFMGLMCC017), and *Aspergillus terreus* (UFMGLMCC032)			
Brazil	Furna dos Morcegos cave	*Aspergillus sydowii* (CML2967)	Not informed—strain from Culture Collection	Antioxidant activity and phenolic compounds	[52]
	Coroa de Frade cave	*Aspergillus keveii* (CML2968)			
	Lapa nova cave	*Aspergillus aureolatus* (CML2964) and *Penicillium flavigenum* (CML2965 and CML3827)			
	Honorato cave	*Epicoccum nigrum* (CML2971)			
	Cavalinho cave	*Lecanicillium aphanocladii* (CML2970)			
Algeria	Chaabe Cave	*Penicillium* spp.	ITS and β tubulin (nBLAST) and morphology	Antimicrobial activity against Gram-positive and Gram-negative bacteria	[43]
Brazil	Cavalinho cave	*Lecanicillium aphanocladii* (CML2970)	Not informed—strain from Culture Collection	Modulating action on proteases and phospholipases A2 present in snake venoms of the *Bothrops* genus	[53]
Brazil	Catão cave	*Aspergillus ustus* (CBMAI 1894), *Talaromyces brunneus* (CBMAI 1895), and *Aspergillus* sp. (CBMAI 1926)	Phylogenetic analysis based only β-tubulin gene and morphology	Cellulolytic activities, endoglucanases, and β-glucosidases	[54]
Iran	Darband cave	*Penicillium chrysogenum* (DDFCC170, DDFCC186, and DDFCC114)	Morphology	Sorbicilin-producing (antimicrobial activity) against *Escherichia coli* (ATCC 25922), *Shigella* (sp.), *Staphylococcus aureus* (ATCC25923), *Bacillus subtilis* (ATCC 12711), *Bacillus cereus* (PTCC1015), *Proteus* (sp.), *Pseudomonas aeruginosa* (ATCC 27853), and *Klebsiella pneumonia* (ATCC13883)	[55]
Brazil	Gruta Toca Cora de Frade	*Aspergillus keveii* (CML2968; ONI75)	Phylogenetic analysis based on ITS region	Pigment production	[56]
	Gruta Lapa Nova	*Penicillium flavigenum* (CML2965)			
	Honorato cave	*Epicoccum nigrum* (CML2971)			
Czech Republic	-	*Pseudogymnoascus* sp. (CCF5025)	ITS (nBLAST)	Elastases and colagenases production	[57]
	-	*Pseudogymnoascus* sp. (CCF5030)			
	Javoříčske Caves	*Pseudogymnoascus* sp. (CCF5027 and CCF5029)			
	Sloupsko-Šošůvké Cave	*Pseudogymnoascus* sp. (CCF5026)			
Spain	Altamira Cave	*Conidiobolus thromboides* (H18) and *Gliomastix murorum* (H2)	ITS, D1/D2 region, mtSSU, SSU, RPB2 (nBLAST)	Laccase-like production	[58]
Sri Lanka	Sthreepura Cave—Kuruwita	*Aspergillus fumigatus* (SKW 301 and SKW 404) and *Trichoderma yunnanense* (SKW 407)	ITS (nBLAST) and morphology	Antimicrobial activity against *Staphylococcus aureus*	[59]
Republic of Serbia	Cave Church of Sts. Peter and Paul	*Penicillium samsonianum* (BEOFB11211m)	ITS and β tubulin (nBLAST) and morphology	Acid production with carbonate dissolution and proteolytic activity	[60]
		*Cladosporium cladosporioides* (BEOFB18216m)	Morphology	Acid production with carbonate dissolution and proteolytic activity	
		*Alternaria alternata* (BEOFB218m)		Cellulolytic and hemicellulolytic active	
		*Botryotrichum murorum* (BEOF5701m)		Cellulolytic and hemicellulolytic active	
		*Stereum gausapatum* (BEOFBB1730)		Cellulolytic and hemicellulolytic active	
		*Coprinellus disseminatus* (BEOFB2120)		Hemicellulolytic active	
		*Aspergillus aureolatus* (BEOFB3320m)	ITS and β tubulin (nBLAST) and morphology	Pigment production	
		*Penicillium freii* (BEOFB11230m)		Acid production, proteolytic activity, and pigment production	
		*Stereum gausapatum* (BEOFBB1730)	Morphology	Pigment production	
		*Beauveria pseudobassiana* (BEOFB910m)		Pigment production	
		*Bjerkandera adusta* (BEOFB1605)		Acid production, proteolytic activity, and pigment production	
		*Mortierella alpina* (BEOFB6006m)		Acid production	
		*Penicillium expansum* (BEOFB11132m)	ITS and β tubulin (nBLAST) and morphology	Acid production	
		*Parengyodontium album* (BEOFB6200m)	Morphology	Proteolytic activity	
		*Botrytis cinerea* (BEOFB3105m)		Acid production	
Brazil	Catão cave	*Aspergillus* sp. (SDC28)	Phylogenetic analysis based only β-tubulin gene and morphology	Versicolorin and versiconol cytotoxic activity against ovarian cancer cells OVCAR3.	[61]
Belgium	Limestone cave	*Botryotrichum* sp. (SVS002), *Trichoderma* sp. (SVS008), and *Mortierella* sp. (SVS004 and SVS005)	ITS (nBLAST)	Precipitate CaCO3 on cement paste	[62]
Brazil	Cave GEM-1462	Yeast not identified (GSD101)	Not informed	Antimicrobial, proteolytic, and cellulolytic activity	[33]
India	Badami Caves	*Aspergillus niger* (B9) and *Cladosporium angustisporum* (B2)	Morphology and ITS (nBLAST)	Biodeterioration potential: carbonate solubilization and organic acids (ascorbic acid and fumaric acid)	[63]
Mexico	Tolantongo canyon	*Aspergillus* sp., *Penicillium* sp., *Trichoderma* sp., *Cladosporium* sp., and *Fusarium* sp.	Morphology	Extracellular enzymes: proteases	[64]

**Figure 1:**
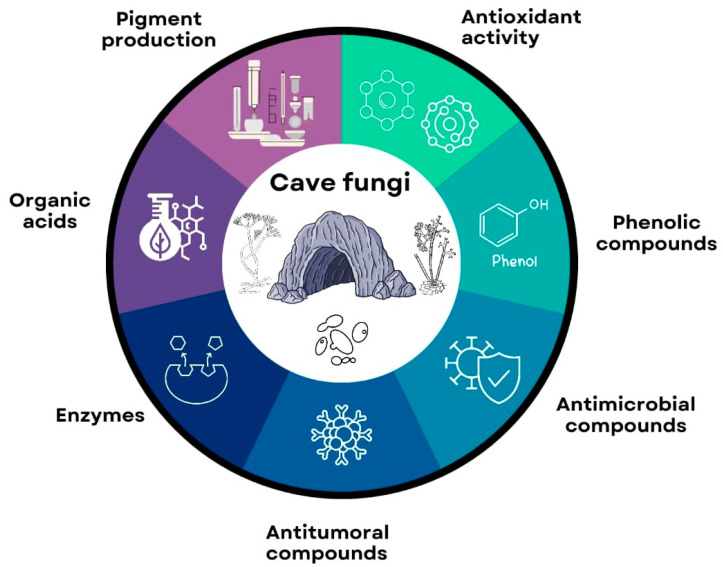
The main biotechnological potential of cave fungi observed in the papers analyzed in this study.

The potential applications were categorized into various topics, such as antimicrobial compounds, antioxidant activity and phenolic compounds, antitumoral compounds, enzymes, organic acids, and pigment production (Figure 1). Our study discusses the main conclusions drawn from these papers in detail. Furthermore, our analysis delved into significant aspects concerning the biotechnological exploration of cave fungi, emphasizing the importance of accurate fungal identification and preservation in culture collections. We highlighted the need for continued research in this area to unlock the full potential of cave fungi for various biotechnological applications.

### 3.1. Antimicrobial Compounds

Microorganisms constitute a valuable source for discovering natural antibiotics, with research focusing on exploring underexplored and extreme environments such as caves [65]. These environments, which are isolated for extended periods, are thus unique and can harbor fungi and other microorganisms with the potential to produce new antimicrobial agents. Furthermore, the oligotrophic conditions and limited resources in caves foster intense competition among microorganisms, enhancing antimicrobial activities [59,66]. These findings have significant implications for the pharmaceutical industry and for production. Recent studies, exemplified by research in the Chaabe cave in Algeria, demonstrated that 23 isolates of *Penicillium* exhibited antifungal activity exceeding 40% against *Candida albicans* [43]. Similarly, in the Sri Lankan Sthreepura Cave, isolates of Aspergillus fumigatus displayed high antibacterial activity against Staphylococcus aureus [59]. Isolates of the genus *Penicillium*, derived from Venezuelan caves, demonstrated antibacterial potential, inhibiting the growth of *Escherichia coli*, *Staphylococcus aureus*, and *Klebsiella pneumoniae* [67]. These findings underscore the cave environment’s richness as a potential source of new antimicrobial compounds with applications in the pharmaceutical industry.

In contrast, Taylor et al. [51] investigated the resistance to antifungals of fungal strains from caves in the iron quadrangle of Brazil. These strains were tested against amphotericin B (AMB), itraconazole, voriconazole, and terbinafine. In total, 32 strains of *Aspergillus candidus*, *A. flavus*, *A. fumigatus*, *A. niger*, *A. tamarii*, and *A. terreus* were analyzed. The minimum inhibitory concentrations (MICs) of terbinafine ranged between 0.003 and 1.0 μg/mL, the MICs of voriconazole ranged between 2.0 and >16.0 μg/mL, the MICs of itraconazole ranged between 0.25 and 8.0 μg/mL, and the MICs of amphotericin B ranged between 0.03 and 4.0 μg/mL. A strain resistant to AMB, identified as *A. flavus*, exhibited an MIC of 4 μg/mL. Resistance to AMB was associated with increased ergosterol levels, enhanced peroxidase and superoxide dismutase enzymatic activity, and reduced lipid peroxidation. These findings provide valuable insights into the mechanisms of antifungal resistance in underground ecosystems, contributing to a better understanding of the microbiota in these unique environments.

### 3.2. Antioxidant Activity and Phenolic Compounds

The incorporation of antioxidant compounds as food additives prolongs the shelf life of products while preserving their nutritional and sensory attributes [68]. Besides their preservative role, these compounds play a crucial part in neutralizing free radicals produced during normal physiological processes in living organisms, helping to mitigate the damage caused by the excessive accumulation of free radicals, which can lead to serious health conditions such as cancer, atherosclerosis, immunosuppression, aging, inflammation, ischemic heart disease, diabetes, and neurodegenerative diseases [69]. Gallic acid, a type of phenolic compound, is known for its potent antioxidant properties, particularly in emulsions and lipid-based systems. It is commonly incorporated into processed foods, cosmetics, and food packaging materials to prevent rancidity caused by lipid oxidation [70]. In addition, gallic acid is used in the pharmaceutical industry for the production of antibiotics and has been shown to exhibit anticancer and anti-inflammatory effects [52]. Therefore, identifying new sources of antioxidant compounds is essential not only for ensuring the durability of food items but also for reducing the harmful impact of free radicals on human health. In a study conducted in Brazil, Tavares et al. [52] investigated the antioxidant potential and phenolic compound composition of extracts from pigment-producing fungi isolated from several caves (see Table 1). The potential of strains of *Aspergillus sydowii* (CML2967), *A. keveii* (CML2968), *A. aureolatus* (CML2964), *Epicoccum nigrum* (CML2971), *Lecanicillium aphanocladii* (CML2970), *Penicillium flavigenum* (CML2965), and *P. flavigenum* (CML3827) was evaluated. The results showed that the ethyl acetate extract from *P. flavigenum* (CML2965) exhibited significant antioxidant activities of 98.2%, 47.1%, and 72.2% according to the DPPH, ABTS•+, and β-carotene assays, respectively. Moreover, the extract exhibited a high content of total phenolic compounds, measuring 201 mg gallic acid equivalent (GAE)/g. In addition to its antioxidant effectiveness, the study highlighted the diversity of phenolic compounds, including catechin, chlorogenic acid, and caffeic acid. Catechin, for example, is a potent free radical scavenger and has the ability to prevent the oxidation of LDL cholesterol, along with possessing anti-inflammatory, antibacterial, and anticancer properties. Meanwhile, chlorogenic and caffeic acids exhibit antioxidant, antimutagenic, and anticancer activities, which expands the therapeutic potential of these compounds [52]. The presence of these substances broadens the range of possible applications for the extract and deepens our understanding of the multifaceted potential of the fungi in question. This could be a critical aspect for future research on the exploitation of cave microorganisms in the development of new bioactive products.

### 3.3. Antitumoural Compounds

Chemotherapeutic drugs have been a mainstay in cancer treatment for several decades, yet many of them have significant cellular toxicity. In light of these concerns, there has been a shift towards utilizing natural anticancer compounds sourced from fungi. These compounds have been isolated and purified, demonstrating efficacy against various cancers, including Kaposi’s sarcoma and prostate, lung, ovarian, and breast cancer [71,72]. For example, two anthraquinones derived from liquid cultures of *Aspergillus* sp. (SDC28) isolated from soil in Catão Cave in Brazil, were identified [61]. These compounds exhibited notable cytotoxic effects on the human ovarian cancer cell line OVCAR3. They obtained IC50 values of 0.24 μM for versicolorin C and 1.07 μM for versiconol, using Taxol as a control, showed an IC50 of 7.0 ± 2 nM. Additionally, the study provided comprehensive spectroscopic characterization, including determination of the absolute configuration of both versicolorin C and versiconol, marking the first such report.

### 3.4. Enzymes

Enzymes produced by fungi are essential for overcoming the host’s natural defenses (pathogenic process) and for generating soluble substances that can be absorbed and utilized as nutrition [48]. In cave fungi, extracellular enzyme production (e.g., amylases, proteases, and cellulases) was assessed by Ogórek et al. [48] using a strain of *Penicillium glandicola* (UWR 002) obtained from sediments in a cave in Slovakia. The authors also observed that this strain lacked the ability to produce pectinases and keratinases under the tested conditions. Additionally, the strain survives for 56 days across a broad range of very low temperatures (from −72 °C to 5 °C), with growth occurring at temperatures ranging from 5 °C to 25 °C. However, no spore germination or active growth was observed at 30 °C or 37 °C. Pectinases cause modifications in the cell wall, increasing the accessibility of its components for degradation by other enzymes, resulting in cell lysis and tissue maceration. Keratinophilic activity is particularly important for fungi that cause superficial fungal infections, such as dermatophytes. Therefore, due to the lack of production of these enzymes, the authors suggested that the fungal species or isolate is likely non-pathogenic to plants, humans, or other animals [48].

Microbial proteases are a crucial class of hydrolytic enzymes that have been extensively researched due to their significant biotechnological potential in a variety of industrial applications, including detergent, textile, leather, dairy, and pharmaceutical preparations. Fungal proteases, in particular, are of interest due to their ability to thrive on cost-effective substrates and secrete substantial quantities of enzymes into the culture medium [73]. In Mexico, Legorreta-Castañeda et al. [64] investigated the diversity of thermotolerant fungi found in caves and hot springs, focusing on their potential for extracellular enzyme production, particularly proteases. This study characterized the morphological diversity of the isolated fungi, identifying genera such as *Aspergillus*, *Penicillium*, *Trichoderma*, *Cladosporium*, and *Fusarium*. A screening process on solid media revealed that 20 out of 35 isolated fungi exhibited proteolytic activity, with 12 strains identified as proficient protease producers. Additionally, the study investigated the formation of fungal pellets by proteolytic fungi, observing significant pellet formation in certain strains. In submerged cultures, the investigation of protease production by fungal pellets revealed one isolate as the highest enzyme producer [64]. These authors also demonstrated the potential of these fungi for the production of amylase, chitinase, and chitosanase enzymes in solid media. Extracellular enzymes produced by thermotolerant and thermophilic fungi exhibit greater catalytic efficiency than those from mesophilic fungi. The use of these enzymes presents several benefits, especially in the food industry, where higher processing temperatures can be applied. These temperatures are linked to faster reaction rates, improved solubility of reagents, and a lower risk of mesophilic contamination [64].

Tannases hold significant industrial importance and have applications in various sectors, including the food, animal feed, cosmetics, pharmaceutical, chemical, and leather industries [74]. In Brazil, Melo et al. [47] used various fungal species isolated from caves and observed that among the 544 fungal strains obtained and evaluated, 386 were capable of growing on media containing tannic acid as the sole carbon source. A total of 32 strains were identified as proficient tannase producers. The highest tannase activity in submerged fermentation was achieved by *Aspergillus japonicus* 246A (16.45 U/mg) and *Aspergillus tamarii* 3 (12.95 U/mg). An important step in biotechnological assays involving many strains of fungi to be tested is the screening of strains capable of producing the enzyme in large quantities and with desirable characteristics for industrial application. The authors conducted a selection by assessing the degradation of carbon sources on solid media, and the isolates that presented larger degradation halos (the light-colored area around the fungal colony) were evaluated for tannase production in submerged fermentation. They observed that there was no direct relationship between the diameter of the halo and tannase production in liquid media. Therefore, they recommended that, in the screening phase, a larger number of microorganisms be assessed and that a quantitative stage be incorporated into the process [47].

Laccases are the oldest and most studied enzymatic systems [75], exhibiting widespread distributions across higher plants, bacteria, fungi, and insects [76,77]. These enzymes have significant industrial applications in sectors such as food [78], paper and pulp [79,80], textiles [66], synthetic chemistry [81], bioremediation [82], and others. Laccase production was investigated by Fernández-Remacha et al. [58] using fungi isolated from a cave in Spain. The authors identified *Gliomastix murorum* (H13) and *Coniodiobolus thromboides* (H18) as laccase producers. The secretion of laccase-like enzymes by both fungi was confirmed through zymography, and additional assays demonstrated the ability of the laccases to degrade industrial dyes, such as Congo Red, Indigo, and Eriochrome Black T. Additionally, advanced techniques such as mass spectrometry and homology modeling used by these authors provided a more detailed understanding of the laccases produced by *G. murorum* and *C. thromboides*. The identification of their functional domains, along with the prediction of their interactions with industrial dyes, offers valuable insights for optimizing biotechnological processes focused on decolorization and degradation of pollutants [58]. These advancements further highlight the potential of these laccases in industrial applications, such as in the textile industry and wastewater treatment.

Cave fungi have also been reported to produce other enzymes, such as cellulases, which are important in various industrial sectors, including biofuel production, food and beverages industries, and agriculture [83]. For example, in a semiquantitative analysis of cellulase production by fungi isolated from Gruta do Catão, Paula et al. [49] reported that among 20 isolates tested, 18 showed enzymatic activity. Notably, *Penicillium* SDC 2.13 (IE 2.40), *Scopulariopsis* SDC 2.1 (IE 2.46), and *Talaromyces* SDC 2.9 (IE 2.48) exhibited significant activity. Cellulolytic enzymes in fungi are activated following the decrease in bacterial biomass caused by the depletion of these carbohydrates, thereby enabling the fungi to dominate the remainder of the cellulose–waste decomposition. Other factors also enable fungi to be more successful than other microorganisms in cellulose decomposition, such as their filamentous growth [49]. The cellulolytic enzyme production observed in 18 of the 20 isolates indicated that these fungi play an essential role in maintaining and enhancing the quality of the subterranean environment, aiding in the recycling of carbon and other nutrients. As the cave environment is oligotrophic and carbon sources are limited, the ability of fungi to utilize cellulose as an energy source could be crucial for the sustainability of this ecosystem [49].

### 3.5. Organic Acids

Shifting to another context, the growth of fungi on mineral surfaces initiates the biological degradation of rocks, involving physical particle separation and the release of secondary metabolites and organic acids [45,84]. These metabolites play a pivotal role in altering the pH of the microenvironment, influencing ion energy on the rock surface. This dissolution process not only provides nutrients but also creates a favorable environment for the growth of other microorganisms associated with minerals [85].

Agrawal et al. [63] investigated the diversity, distribution, and biodeterioration phenomena caused by fungi in archaeological carvings located in the Badami Caves, India. Using specialized techniques, they monitored calcite dissolution, changes in pH levels, and the biomineralization capabilities of the isolated fungal strains. Additionally, the production of fungal acids was analyzed through high-performance liquid chromatography (HPLC). The authors observed that the fungi were *Acremonium*, *Curvularia*, *Cladosporium*, *Penicillium*, and *Aspergillus*. Two isolates, *Cladosporium angustisporum* (B2) and *Aspergillus niger* (B9), exhibited the formation of transparent zones around their colonies growing on CaCO_3_ glucose agar medium, indicating their ability to dissolve CaCO_3_. *Aspergillus niger* (B9) showed the most significant change in pH, decreasing from 7.5 (control value) to 2.24 ± 0.24. They also observed evidence of the production of ascorbic acid and fumaric acid by *Cladosporium* sp. (B2) and citric acid and fumaric acid by *Aspergillus niger* (B9). These findings provide valuable insights into the ecology and functions of fungi inhabiting stone surfaces, enhancing our understanding of biodeterioration processes and contributing to the development of strategies for preserving and protecting sculptures.

### 3.6. Pigment Production

There is a growing interest in substituting toxic synthetic colorants with natural alternatives. Microorganisms, particularly filamentous fungi, are considered promising sources of pigments due to their predictable and controllable yield, with applications in various industries [86,87,88]. Filamentous fungi isolated from Brazilian caves exhibit the potential for producing natural colorants, which are already being used in the food sector and other industries. Souza et al. [56] assessed the production of pigments through submerged fermentation by three filamentous fungi isolated from Brazilian caves: *Aspergillus keveii* (CML2968), *Penicillium flavigenum* (CML2965), and *Epicoccum nigrum* (CML2971). The resulting pigments were subjected to spray drying with adjuvants such as maltodextrin, modified starch, or gum arabic. The process successfully produced yellow fine powders with low moisture content, low water activity, and high color retention (>70%). Regardless of the adjuvant used, the dried products demonstrated enhanced stability, a high product recovery ratio (>50%), and potential for use as natural colorants in food and pharmaceutical applications.

### 3.7. Cave-Specific Conditions Influence the Fungal Production of Bioactive Compounds

The conditions of cave environments significantly influence the metabolic strategies of microorganisms, enabling them to survive and serve as valuable sources of various bioproducts [89]. In particular, fungal species can produce bioactive compounds in response to these unique cave conditions. For example, darkness plays a critical role in fungal metabolism, influencing the production of conidia [90], pigmentation [91], and metabolites [92].

The pH of cave environments further influences microbial distribution and metabolism. Many fungi are adapted to specific pH conditions, ranging from acidic to alkaline [93,94]. In caves with acidic conditions, microbial populations are often dominated by species that thrive in low-pH substrates. For example, *Acidomyces acidophilum*, isolated from acidophilic biofilms in the Sheki-Heh Cave (Chechen Republic of the Russian Federation), is well adapted to such environments [95]. These adaptations enable fungi to metabolize efficiently in such environments, and their metabolism can even contribute to the precipitation of minerals like calcium carbonate (CaCO_3_), which is influenced by the local pH levels. Enzymes synthesized by acidophilic fungi are particularly important, as they exhibit efficient catalytic activity under adverse conditions in various industrial processes where enzymatic functions are typically unfavorable [96]. For example, xylanase from the acidophilic fungus *Bispora* sp. MEY-1 demonstrated peaks of enzymatic activity at pH 3.0 and 4.5–5.0 (100%) and retained more than 80% of its initial activity after incubation at pH values ranging from 1.5 to 6.0 for 1 h [97].

In caves hosting bat species, guano is a crucial carbon source for fungal species. For instance, insectivorous guano is rich in carbon and nitrogen on its surface, with the carbon-to-nitrogen ratio decreasing with increasing depth [98]. This demonstrates a variance in the availability of these nutrients and may influence the abundance and distribution of fungi capable of surviving in such conditions [99]. Nitrogen and carbon sources regulate several secondary metabolism genes in fungal species [100]. Lockington et al. [101] found that the production of cellulases by *Aspergillus nidulans* strains was influenced by the nitrogen and carbon source, with some of these enzymes regulated by the global nitrogen metabolism regulator (*AreA*) and by carbon metabolism regulators (*CreA*, *CreB*, and *CreC*).

Average cave temperatures vary depending on the climate zones in which they are located, exhibiting either lower or higher temperatures. In general, temperatures in caves tend to be more stable than on the surface, with less fluctuation. Distinct patterns of correlation between temperature variability in caves and on surfaces have been observed, indicating that surface conditions influence the average annual temperature of caves but also depend on the specific characteristics of each cave [102]. These temperature variations can influence the metabolic potential of microorganisms inhabiting these environments [103]. Psychrophilic and psychrotrophic fungi, for example, are important in biotechnology and pharmaceuticals due to the adaptations that allow them to survive in extreme conditions [104]. Nascimento et al. [105] showed that the protease enzyme synthesized by the psychrophilic fungus *Acremonium* sp. L1-4B demonstrated thermal stability for 180 min at temperatures ranging from 10 °C to 40 °C.

Interestingly, some caves are rich in iron and are often referred to as ferruginous caves. These environments are characterized by the presence of iron-rich minerals, which significantly influence the microbial communities that thrive there. The availability of iron in these caves provides an important resource for fungi, further shaping their metabolic processes and the production of bioactive compounds. Iron plays a crucial role in various metabolic pathways, such as cellular respiration, DNA biosynthesis, and the activation of enzymes responsible for electron transfer [106]. Its availability can promote fungal growth by enhancing ATP production and optimizing iron-dependent functions. However, regulating iron levels is vital, as both iron deficiency and excess can harm the cell, causing delays in the cell cycle or oxidative damage [107]. Additionally, iron influences the production of bioactive compounds, such as laccases, which are important for fungal metabolism and adaptation in environments with varying concentrations of this metal. For instance, Hernández-Monjaraz et al. [108] evaluated the importance of iron in laccase production in *Fusarium oxysporum* f. sp. *Lycopersici* strain *rho1::hyg* showing that the addition of iron chelators increased enzyme activity in the intracellular fractions, highlighting its role in regulating bioactive compound production in fungi.

It is important to highlight that although the studies reviewed provided valuable insights into the biotechnological potential of fungi isolated from caves, the environmental conditions of these caves were not assessed in relation to the production of the metabolites discussed in this review. Thus, there remains a research gap that could be explored to better understand how specific cave environmental factors, such as pH, temperature, and the availability of nutrients and metals, collectively influence the biosynthesis of fungal metabolites.

### 3.8. Why Is It Important to Investigate the Biotechnological Potential of Cave Fungi?

The biotechnological potential of cave fungi is significant due to the unique ecological niche created by the cave environment [43]. Characterized by extreme oligotrophy and low organic carbon concentrations, caves foster the development of highly specialized microorganisms [85,109,110]. The resource limitation in caves drives intense competition among microorganisms, leading to remarkable metabolic bioactivity and the production of substances with inhibitory potential against other microorganisms [33,59].

Direct comparisons with other ecosystems have become challenging due to the unique selective forces in caves that influence microorganisms [44,45,46]. Evolutionary pressure in this environment prompts cave fungi to develop new compounds or metabolic pathways with significant biotechnological interest [45,85]. The scarcity of nutrients in cave ecosystems promotes microbial competition, leading them to evolve survival strategies, including the secretion of metabolites such as antibiotics and enzymes [22].

The pharmaceutical industry is increasingly looking to natural sources, like fungi, for potential new antimicrobial agents due to the limitations of existing drugs and the rise in antibiotic resistance [111,112]. Fungi from cave environments can represent an exceptional opportunity for this exploration. The oligotrophic conditions in caves foster intense microbial competition, which can enhance the production of antimicrobial compounds. Studies have shown that isolates from caves, such as those from the Chaabe Cave in Algeria and Sthreepura Cave in Sri Lanka, exhibit significant antifungal and antibacterial activities, highlighting their potential as sources for new drugs [43,59].

Furthermore, cave fungi have the potential to create antioxidant and anticancer chemicals in addition to being interesting sources of antibacterial agents. Fungi-derived antioxidants can lessen the negative effects of free radicals, which have been connected to a number of illnesses, including cancer [69]. Studies on fungi found in Brazilian caves have revealed high phenolic content and notable antioxidant activity in some strains, demonstrating its potential for biotechnology [52]. Furthermore, anthraquinones extracted from cave fungi have shown cytotoxic effects on human ovarian cancer cell line OVCAR3, suggesting that they may be used to treat cancer [61].

The unique ecological niches provided by caves allow for the development of specialized fungal strains that may produce enzymes with diverse applications. For example, extracellular enzymes such as amylases and proteases, which are used in industrial operations ranging from food production to pharmaceuticals, have been demonstrated to be produced by cave fungi [73]. These fungi have developed the ability to produce such enzymes because of the evolutionary stresses and resource scarcity seen in cave habitats, which force them to create metabolic pathways [45]. Thus, investigating cave fungi for novel biomolecules increases our knowledge of microbial ecology while also being essential for tackling urgent health and industrial issues. Under special circumstances, these fungi may produce new molecules that could result in important breakthroughs in biotechnology and medicine.

### 3.9. Accurate Cave Fungal Identification Is Crucial in Biotechnology

Fungal identification is critical in mycological studies, as it is based on a combination of morphological, morphological, phylogenetic, or ecological characteristics. Classical methods for fungal identification involve direct observation, either in their natural state or after cultivation on growth media. Integrating molecular methods with morphological studies has proven invaluable, enabling mycologists to examine new fungal samples or reevaluate preserved ones and leading to the proposal or establishment of many new taxa [113].

Accurate fungal identification is essential for scientific research and applications in ecology, taxonomy, genomics, and bioprospecting [114,115]. Scientific names play a pivotal role in conveying information about fungi, aiding researchers in predicting the evolution of chemical gene clusters and prioritizing taxonomically related strains for optimal bioactive compound production.

Fungal taxonomy, traditionally based on morphological characteristics, is undergoing a revolution with the integration of molecular methods, making it increasingly important for accurate classification [116,117,118]. Morphology-based and molecular data-based methods are employed in fungal identification, each offering complementary insights into the classification process Integrating morphological approaches with chemical, ecological, molecular, or physiological analyses has become crucial [113].

During our literature survey, several fungal strains evaluated for biotechnological potential were identified solely based on morphological features and/or through BLASTn comparisons in the GenBank database, raising concerns about inaccuracies in nomenclature. Misidentifications, especially within genera such as *Penicillium* and *Aspergillus*, are a significant challenge. This raises the concern of potential misclassification of fungal strains solely by relying on a BLASTn search. It is crucial that the identification of fungi is performed correctly. For instance, species closely related to *A. niger*, such as *A. neoniger*, *A. tubingensis*, *A. luchuensis*, *A. vadensis*, and *A. tubingensis* are employed in various industry segments, underscoring the critical importance of accurate identification. Misidentifications of these species, particularly when misclassified as *A. niger*, have been documented and highlight the need for careful and precise identification in both scientific research and industrial applications [118].

**Figure 2:**
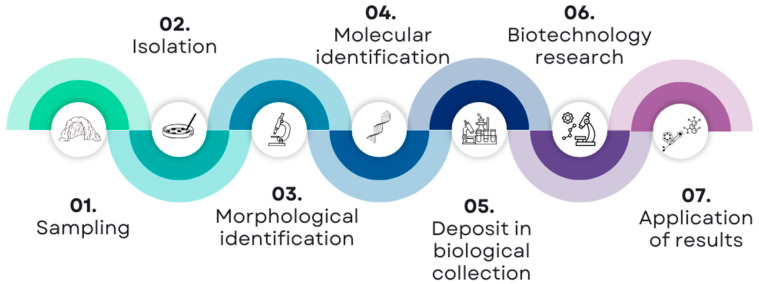
Proposed workflow for working with fungi isolated from caves, from sampling to biotechnological applications.

Fungal taxonomy is complex, and the identification of fungal species presents significant challenges for biotechnologists. Fungal identification tools have evolved considerably, from phenetic methods to genome sequencing. Although the ITS region is often regarded as a barcode for fungi, relying solely on it for species identification may not be optimal for various fungal groups. Furthermore, due to clade-specific evolutionary histories, there is currently no single tool available for the identification of fungi [119]. Species identification often requires additional genes or regions. For fungi that are evaluated for biotechnological potential, be they cavernous fungi, as well as those isolated from other substrates and environments, we emphasize the importance of conducting phylogenetic analyses using sequences from type materials for precise species identification rather than solely relying on simplistic similarity searches via tools such as BLASTn on the NCBI platform. In Figure 2, we proposed a workflow for working with fungi isolated from caves, from sampling to biotechnological application.

The importance of accurate fungal identification in biotechnology can be summarized in three key reasons. (1) The scientific name provides essential information about a fungal species’ biology, ecology, and evolutionary relationships. (2) Accurate fungal identification is indispensable for preventing the misuse of strains with pathogenic potential or the selection of undesirable species, especially in the food industry, where a preference exists for microorganisms generally recognized as safe (GRAS). (3) The precise identification of fungi is vital for advancing our understanding of fungal biology. In speleomycology, recent studies have demonstrated the utility of various identification tools, including traditional morphology-based methods and advanced phylogenetic analyses, which have provided new insights into fungal diversity in caves [28,29,30,31,32,33,34,120]. These findings not only enhance our understanding of fungal taxonomy but also open doors for potential biotechnological innovations derived from cave fungi.

### 3.10. Explore, Study, and Preserve Cave Fungi

For a long time, biodiversity conservation and biotechnology have traditionally been opposite poles of biological sciences, but this scenario has been increasingly changing due to the broad vision of current professionals and the tools and technologies available [3]. Microbiological studies in cave environments should not be confined to biodiversity analysis but should also serve as a tool to guide the development of policies focused on the protection, conservation, and sustainable use of underground environments. An example of this approach can be found in the management plans of caves in Brazil (Caverna Furna Nova/Plano de Manejo Espeleológico—Portaria ICMBio N° 1074 e Caverna dos Crotes/Licença Simplificada—N° 2023-197885/TEC/LS-0317). The development of these management plans, which includes guidelines for use and visitation, encourages the establishment of public policies to protect these ecosystems and, consequently, the fungi and other organisms inhabiting these ecosystems. Protecting these underexplored environments is crucial due to their ongoing potential to yield microorganisms that could lead to the discovery of novel compounds, significantly advancing scientific knowledge [43,44].

In this way, culture collections play a pivotal role in sustainability by utilizing and conserving microbial diversity [121,122]. These collections act as multifaceted repositories, housing biological and genetic materials essential for taxonomic research, biodiversity conservation, and diverse biotechnological applications. They are critical for safeguarding microbial diversity against the gradual environmental erosion caused by global changes and human activities, which can imperil the vital roles of microorganisms in ecosystem functioning [123].

Looking forward, the deposit of fungi in recognized public reference collections, such as those registered with the World Federation for Culture Collections (WFCC) (https://wfcc.info/home_view), is crucial. Several institutions, such as the URM culture collection at Federal University of Pernambuco in Brazil (https://www.ufpe.br/micoteca), the CBS collection at the Westerdijk Fungal Biodiversity Institute in the Netherlands (http://www.wi.knaw.nl/Collections), and the Micoteca of the University of Minho (MUM) in Portugal (http://www.micoteca.deb.uminho.pt/en/), are great examples and recommended for depositing fungal isolates with different potentials [124]. This ensures preservation and enhances the accessibility and widespread utility of these mycological resources in various sectors, including pharmaceuticals, food, agriculture, and cosmetics. It is advisable to deposit fungi in recognized public reference collections listed by the WFCC to benefit the scientific community and applied sectors.

**Figure 3:**
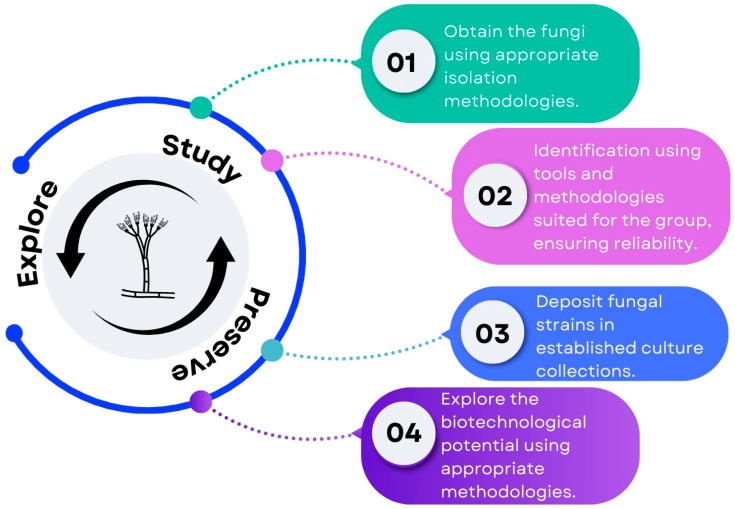
Essential steps for a correct fungal biotechnological exploration.

To guide biotechnological exploration using correctly identified fungal strains, we propose a flowchart (Figure 3) encompassing essential steps for a correct fungal biotechnological exploration. This systematic approach ensures that biotechnological endeavors are conducted with accuracy and reliability.

Given the importance of the topic, we encourage all researchers to obtain fungal strains (from caves or other environments/substrates) in compliance with the legal regulations of their country so that they can isolate, identify, and conduct biotechnological assays, following current methods and widely accepted practices within the scientific community. Researchers are also encouraged to deposit fungi in public repositories in the same way that DNA sequences and proteins are deposited in accessible databases. This is particularly important for fungal strains used in biotechnology and genomic sequencing projects. For instance, the correlation between genomic information and physiological traits would be impossible if the fungus is not preserved in a collection. By ensuring the availability of these strains to other researchers, we also contribute to the global scientific knowledge base and foster greater innovation within the field of fungal biotechnology.

## 4. Conclusions

Our study provides a bibliographic review of the biotechnological potential of fungi from caves worldwide. The biotechnological potential of fungi from caves remains overlooked, and the main biotechnological aspects evaluated to date are the production of antimicrobial, antitumoral, and phenolic compounds; antioxidant activity; and the production of enzymes, organic acids, and pigments. Although these cave fungal strains have been explored for biotechnological applications, their identification has been predominantly by morphological analysis. To advance in this field, biotechnologists should adopt more robust methodologies for cave fungal identification. This review highlights the crucial need to protect little-explored cave environments and microorganisms. The understanding and protection of underground environments contribute to expanding scientific knowledge and are valuable opportunities for innovation in industry and related fields. This review provides a thought baseline for exploring cave fungi and their metabolites for biotechnology and industrial uses.

## Data Availability

Not applicable.
